# Effect of Gypsum on the Early Hydration of Cubic and Na-Doped Orthorhombic Tricalcium Aluminate

**DOI:** 10.3390/ma11040568

**Published:** 2018-04-07

**Authors:** Ana Paula Kirchheim, Erich D. Rodríguez, Rupert J. Myers, Luciano A. Gobbo, Paulo J. M. Monteiro, Denise C. C. Dal Molin, Rui B. de Souza, Maria Alba Cincotto

**Affiliations:** 1 Department of Civil Engineering of Federal, University of Rio Grande do Sul (UFRGS), Porto Alegre 90035-190, Brazil; erich.rodriguez@ufsm.br (E.D.R.); dmolin@ufrgs.br (D.C.C.D.M.); 2 Department of Structures and Civil Construction, Federal University of Santa Maria (UFSM), Santa Maria 971015-900, Brazil; 3 School of Engineering, University of Edinburgh, King’s Buildings, Sanderson Building, Edinburgh EH9 3BF, UK; rupert.myers@ed.ac.uk; 4 Malvern PANalytical, 117 Flanders Rd # 120, Westborough, MA 01581, USA; Luciano.gobbo@panalytical.com; 5 Department of Civil and Environmental Engineering, University of California, Berkeley, CA 1792, USA; monteiro@berkeley.edu; 6 Polytechnic School of the University of São Paulo—POLI/USP, São Paulo 05508-010, Brazil; rui.souza@fei.edu.br (R.B.d.S.); cincotto@usp.br (M.A.C.)

**Keywords:** cubic tricalcium aluminate, orthorhombic tricalcium aluminate, gypsum, hydration, calorimetry, in situ XRD

## Abstract

The tricalcium aluminate (C_3_A) and sulfate content in cement influence the hydration chemistry, setting time and rheology of cement paste, mortar and concrete. Here, in situ experiments are performed to better understand the effect of gypsum on the early hydration of cubic (cub-)C_3_A and Na-doped orthorhombic (orth-)C_3_A. The isothermal calorimetry data show that the solid-phase assemblage produced by the hydration of C_3_A is greatly modified as a function of its crystal structure type and gypsum content, the latter of which induces non-linear changes in the heat release rate. These data are consistent with the in situ X-ray diffraction results, which show that a higher gypsum content accelerates the consumption of orth-C_3_A and the subsequent precipitation of ettringite, which is contrary to the cub-C_3_A system where gypsum retarded the hydration rate. These in situ results provide new insight into the relationship between the chemistry and early-age properties of cub- and orth-C_3_A hydration and corroborate the reported ex situ findings of these systems.

## 1. Introduction

Tricalcium aluminate (Ca_3_Al_2_O_6_ or also known in cement chemistry notation as C_3_A)—the most reactive phase in Portland cement (PC)—begins reacting essentially instantaneously once in contact with water to produce a hydroxy-AFm-type meta-stable product, which is subsequently converted to katoite (Ca_3_Al_2_(OH)_12_ or C_3_AH_6_) and heat. Flash setting can occur if this reaction proceeds unhindered [1,2,3], which is undesirable because it reduces the workability and final strength of the cement paste and, consequently, the mortar and concrete. Other calcium aluminate hydrates (4CaO·Al_2_O_3_·*n*H_2_O or hydroxyl-AFm), e.g., C_2_AH_7.5_ and C_4_AH_x_, are also produced from the hydration of PC clinker in the absence of added calcium sulfate. Calcium sulfate (typically ~5 wt %) is normally milled with PC clinker to retard the C_3_A hydration rate [4], which leads to longer setting times [5,6,7] and a longer time window in which acceptable workability is achieved. The most common type of calcium sulfate added to PC clinker is gypsum, but anhydrite and hemihydrate can also be used.

Previous research regarding the C_3_A-CaSO_4_·H_2_O system showed that the C_3_A hydration rate is related to its crystal structure and specific surface area, the temperature, water/solid ratio [8], type and amount of CaSO_4_ (gypsum, hemihydrate, anhydrite) [6,9,10,11,12,13,14], the presence of other mineral or chemical admixtures [15,16,17,18,19], and the solution chemistry [20,21].

Minor chemical constituents such as MgO, K_2_O, Na_2_O, or SO_3_ that are present in the raw materials or fuels used in clinker production can significantly modify the types and amounts of the clinker phases produced [1]. Na_2_O is typically incorporated into the C_3_A phase, which modifies its crystal structure from a cubic (cub) to an orthorhombic (orth) type through a Ca for Na substitution [22,23,24,25,26,27]. Although the hydration of cub-C_3_A has been extensively studied over the past several decades [2,3,5,6,8,10,12], research regarding the hydration of orth-C_3_A is much scarcer [28,29,30,31,32,33] with additional work needed to more reliably understand the chemistry of this latter system. It is, however, known that C_3_A hydration is significantly influenced by the presence of Na in its C_3_A structure and thus its crystal polymorph type [29]. Recently, it was reported that the dissolution of Al_6_O_18_^18−^ ring structures is strongly dependent on the C_3_A crystal structure. The Ca/Al_[aq]_ ratio measured by inductively coupled plasma optical emission spectroscopy (ICP-OES) during the first few hours of the reaction showed a lower solubility of cub-C_3_A within a calcium sulfate solution when compared to the orth-C_3_A-based systems. This relatively low solubility plays an important role in the formation of an Al-rich layer at the partially dissolved cub-C_3_A solution interphase and therefore affects the dissolution rate [34].

To consolidate the understanding of the effects that gypsum has on the hydration of cub- and orth-C_3_A, this paper presents an integrated in situ assessment of the chemistry of the (cub- and orth-) C_3_A-CaSO_4_-H_2_O system using isothermal conduction calorimetry (IC) and in situ X-ray diffraction (XRD) over a range of CaSO_4_ concentrations relevant to hydrated PC.

## 2. Materials and Methods

Powder samples of cub- and orth-C_3_A were obtained from CTL, Inc., Skokie, IL, USA. Both compounds were synthesized in a laboratory by heating a stoichiometric blend of reagent grade CaCO_3_ and alumina (Al_2_O_3_). Orth-C_3_A was prepared from reagent grade CaCO_3_, Al_2_O_3_, and Na_2_CO_3_ in stoichiometric proportions, similar to the process reported by Regourd et al. [35]. The blends were fired at 1400 °C for 1 h and then quenched using forced air convection. The powders were then produced by ball milling to obtain particle size distributions with d_90_ < 30 μm and mean particle sizes of 11.0 μm and 14.0 μm for orth- and cub-C_3_A, respectively. Pure gypsum (CaSO_4_·2H_2_O) was used in this study (Fisher Scientific, Hampton, NH, USA), which had a particle size distribution with d_90_ < 39 μm and a mean particle size of 19.0 μm.

**Figure 1:**
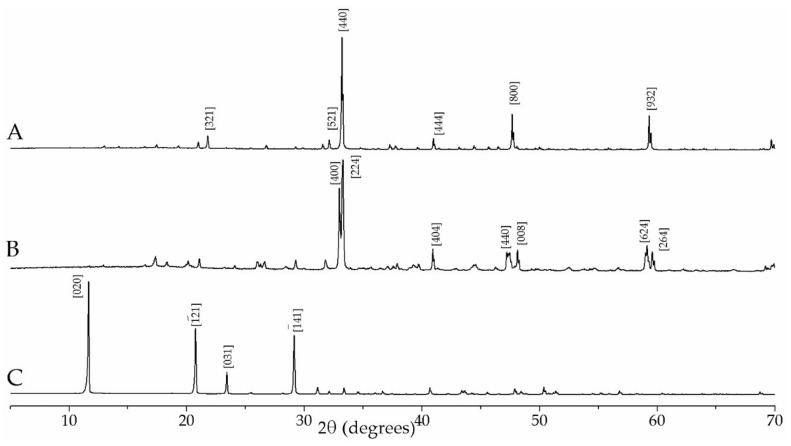
X-ray diffractograms for the un-reacted materials. (**A**) Cub-C_3_A; (**B**) Orth-C_3_A; and (**C**) Gypsum.

The crystal structures of the samples were verified by XRD using a PANalytical X’Pert Pro (PANalytical, Almelo, The Netherlands) (Cu Kα radiation with a step size of 0.02° 2θ). The major diffraction signals of orthorhombic C_3_A International Crystal Structure Database (ICSD) code # 1880 at d-spacings of 2.692 Å and 2.714 Å were clearly identified in the diffractogram of the orth-C_3_A powder, as is shown in Figure 1. The single major reflection of cubic C_3_A (ICSD# 1841) at 2.6987 Å was observed in the diffractogram of the cubic-C_3_A powder. Rietveld analyses using HighScore Plus™ software (Version 4.6, PANalytical, Almelo, The Netherlands) (estimated uncertainty = ±5 wt %) showed that the cub-C_3_A powder contained ≥99 wt % cubic C_3_A (ICSD# 1841) and ~1 wt % C_3_AH_6_ (ICSD# 202316), whereas the orth-C_3_A powder contained ≥92 wt % orthorhombic C_3_A (ICSD# 1880), ~7 wt % C_3_AH_6_ (ICSD# 202316) and ~1 wt % monohydrocalcite (ICSD# 100846, CaCO_3_·2H_2_O).

Samples were produced from mixtures of gypsum and cub- or orth-C_3_A at respective mass ratios of 0, 0.20, 0.60, and 1.90. The gypsum/C_3_A ratio of 1.90 corresponds to the stoichiometry of ettringite formation at a 100% reaction of C_3_A. A water/solid (w/s) mass ratio of 1.2 was used.

In situ XRD: Samples were analyzed using a PANalytical Empyrean diffractometer equipped with an RTMS (real-time multiple strip) detector (X’Celerator) (PANalytical, Almelo, The Netherlands). Materials were weighed and hand mixed inside a plastic bag for 1 min before filling the sample holder, and then immediately covered with a Kapton film to avoid water evaporation and minimize superficial carbonation. A semi-quantitative analysis of the X-ray diffractograms was performed using the reference intensity ratio (RIR) method based on the multi peaks approach as implemented in the High Score Plus™ software. The method involves comparing the intensity of one or more peaks of a phase with the intensity of a peak of a standard material to yield an approximation of the solid phase assemblage in a sample as was discussed by [36]. It was used as an alternative method to get information and a brief comparison based in overall peak intensities from the multiple identified phases. Some of the solid phases produced have a preferred orientation, e.g., ettringite, which can modify the relative intensities of the diffraction signals and the quantitative results. The displacements in the X-ray diffractogram peaks caused by the changing sample volume, a result of the hydration reaction, were corrected by assigning the major ettringite peak to 9.1° 2θ from the observed 2θ value and by shifting the other phases by the same amount using HighScore Plus™ software.

Isothermal calorimetry (IC): The hydration of both cub- and orth-C_3_A was followed using a high-sensitivity (20 μW) isothermal calorimeter (TA Instruments, New Castle, DE, USA) with an integrated stirrer for internal mixing. Samples (3–6 g) of the dry material (gypsum and C_3_A) were first introduced into the cell. Water was added when thermal equilibrium was achieved, and the sample was stirred for 2 min. The evolution of the heat flow produced from the hydration reaction proceeded for 24 h, which began when water was introduced into the cell. The results were normalized to the total mass of solids added.

## 3. Results and Discussion

### 3.1. Hydration of C_3_A without Gypsum—In Situ XRD

**Figure 2:**
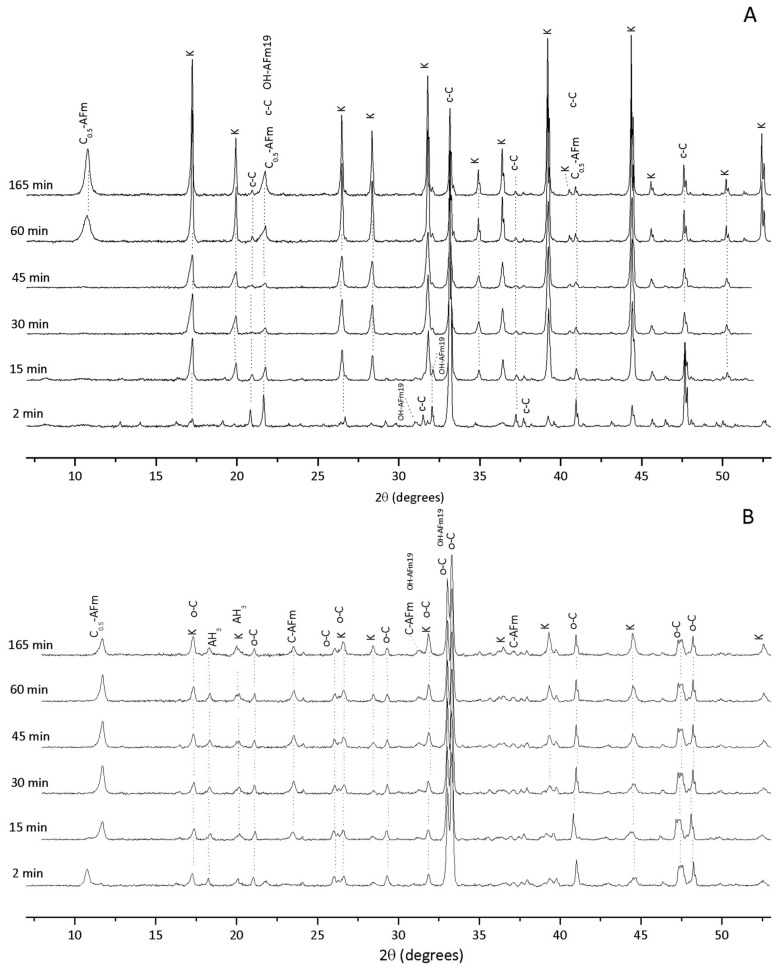
In situ X-ray diffraction (XRD) results for (**A**) cub-C_3_A and (**B**) orth-C_3_A hydration without gypsum. OH-AFm_19_ = C_4_AH_19_; C_0.5_-AFm = calcium hemicarboaluminate hydrate; AH_3_ = gibbsite; k = katoite (C_3_AH_6_); c-C = cub-C_3_A; and o-C = orth-C_3_A.

Figure 2 shows the crystalline hydration products formed between 2 min and 165 min after initiating hydration for cub- and orth-C_3_A without gypsum. The main solid phases produced in both samples are C_4_AH_19_ (OH-AFm_19_; Ca_4_Al_2_O_7_·19H_2_O; powder diffraction file (PDF#) 00-014-0628), calcium hemicarboaluminate hydrate (C_0.5_-AFm; Ca_4_Al_2_O_6_(CO_3_)_0.5_·11.5H_2_O; PDF# 00-041-0221), calcium monocarboaluminate hydrate ($\bar{C}$-AFm, 3CaO·Al_2_O_3_·CaCO_3_·11H_2_O; PDF#01-087-0493), gibbsite (AH_3_; PDF#01-070-2038) and katoite (k; C_3_AH_6_; PDF# 00-024-0217). Cubic C_3_A (c-C; PDF# 00-038-1429) and orthorhombic C_3_A (o-C; PDF# 00-026-0958) were also identified in the samples.

The peaks related to C_3_AH_6_ exhibited high intensities and appeared prior to two minutes of the reaction, which are attributed to the precipitation of this phase as a major hydration product in both cub- and orth-C_3_A systems (Figure 2A,B). The diffraction signals for C_0.5_-AFm occur after 60 min of hydration in the cub-C_3_A system and after two minutes in the orth-C_3_A system. This phase is formed through superficial carbonation of the calcium aluminum hydrate (such as C_4_AH_19_). The presence of calcium monocarboaluminate hydrate ($\bar{C}$-AFm, 3CaO·Al_2_O_3_·CaCO_3_·11H_2_O) and the earlier formation of hemicarboaluminate in the orth-C_3_A system is consistent with the results reported by Dubina et al. [37], showing that orth-C_3_A exhibits a higher susceptibility to superficial carbonation. The results are consistent with previous reports [10], although OH-AFm_19_ is sometimes identified as a major solid hydration product [10,38], but the intensity of the peaks related to its presence were lower.

### 3.2. Hydration of C_3_A with Gypsum—In Situ XRD

**Figure 3:**
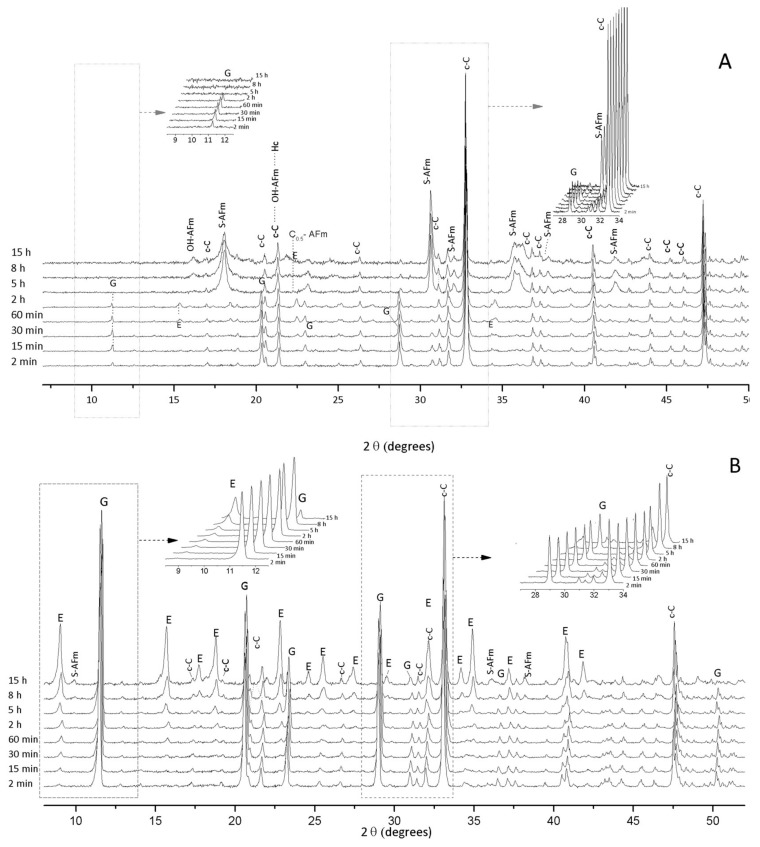

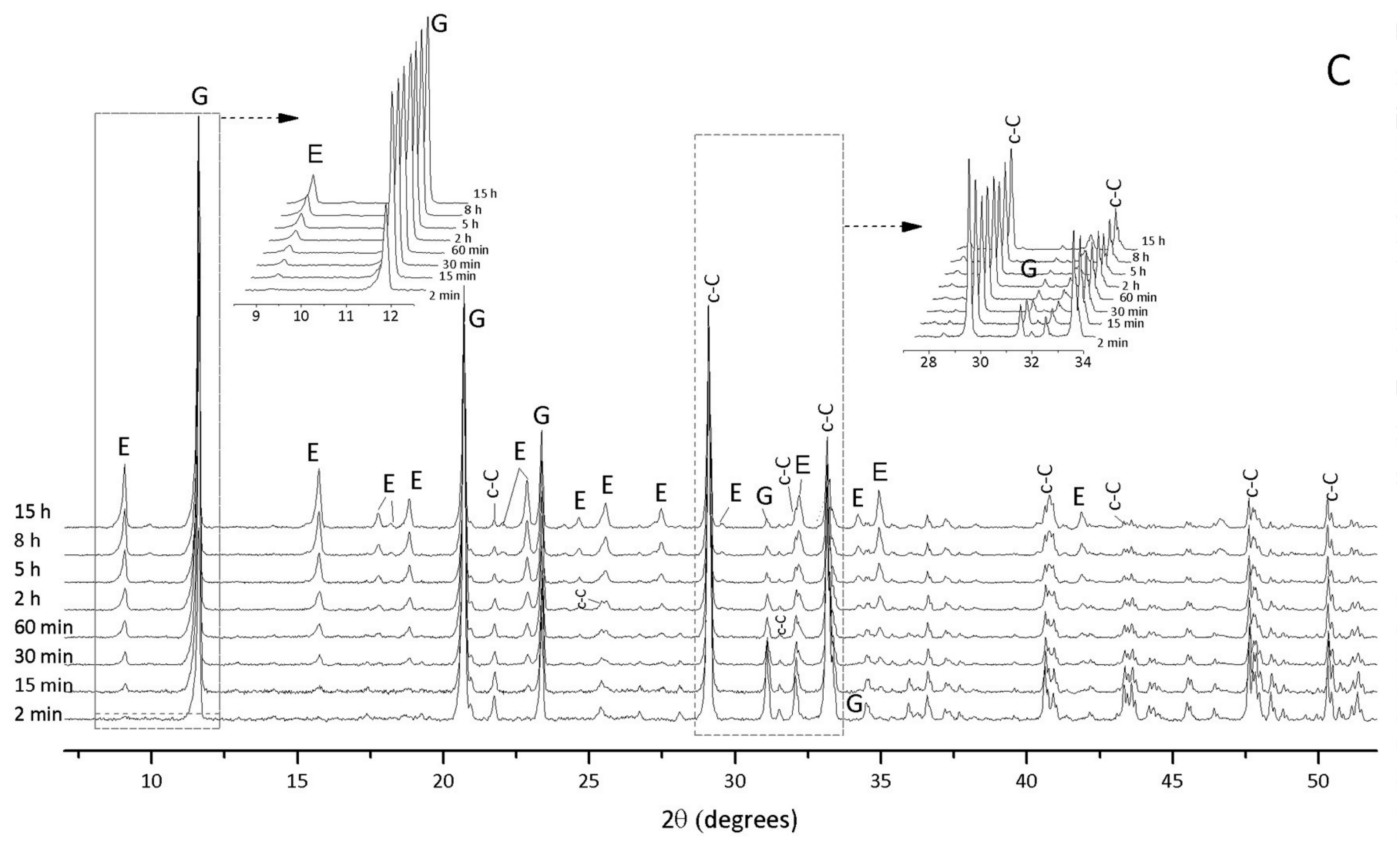
In situ XRD results of cub-C_3_A and gypsum hydration at gypsum/cub-C_3_A ratios of (**A**) 0.20; (**B**) 0.60; and (**C**) 1.90. c-C = cub-C_3_A, E = ettringite, G = gypsum, OH-AFm = C_4_AH_19_; C_0.5_-AFm = hemicarboaluminate; $\bar{S}$-AFm = monosulfoaluminate.

Figure 3 presents the in situ XRD results of cub-C_3_A and gypsum hydration at gypsum/cub-C_3_A ratios of 0.20, 0.60, and 1.90. A fast depletion of gypsum was identified for the cub-C_3_A system with 20% of gypsum after approximately two hours (Figure 3A), and after five hours some unreacted cub-C_3_A remained. The total consumption of gypsum for this sample led to the formation of calcium monosulfoaluminate hydrate ($\bar{S}$-AFm, Ca_4_Al_2_O_6_(SO_4_)·14H_2_O; PDF# 00-042-0062) at the expense of ettringite (E, Ca_6_Al_2_(SO_4_)_3_(OH)_12_·26H_2_O, C_6_A$\bar{S}$_3_H_32_; PDF# 00-041-1451)), as expected. Peaks attributed to ettringite exhibited high intensities for the cub-C_3_A systems with higher contents of gypsum (CaSO_4_·H_2_O; G; PDF# 00-033-0311). $\bar{S}$-AFm was identified mainly for gypsum/cub-C_3_A ratios of 0.20, which is consistent with previous reports [5], where the dissolution of ettringite and the remnant cub-C_3_A formed monosulfoaluminate and/or hydroxy-AFm phases. This reaction occurs after the depletion of gypsum, which is also coherent with the heat released, as is shown below. After eight hours of hydration time, OH-AFm_19_ was detected. Cub-C_3_A was not fully consumed even after 15 h of hydration regardless of the gypsum content.

For the systems, gypsum/cub-C_3_A = 0.60 and 1.9, ettringite is the first crystalline phase identified after two minutes of hydration. Unhydrated gypsum and cub-C_3_A are still present at the end of the measurements (15 h) but exhibit peaks with smaller intensities in the gypsum/cub-C_3_A = 0.6 sample. For the system gypsum/cub-C_3_A = 0.6, the precipitation of $\bar{S}$-AFm began after eight hours.

**Figure 4:**
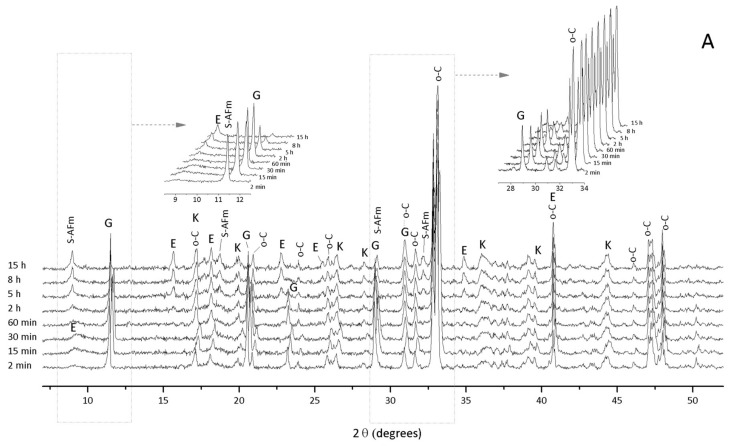

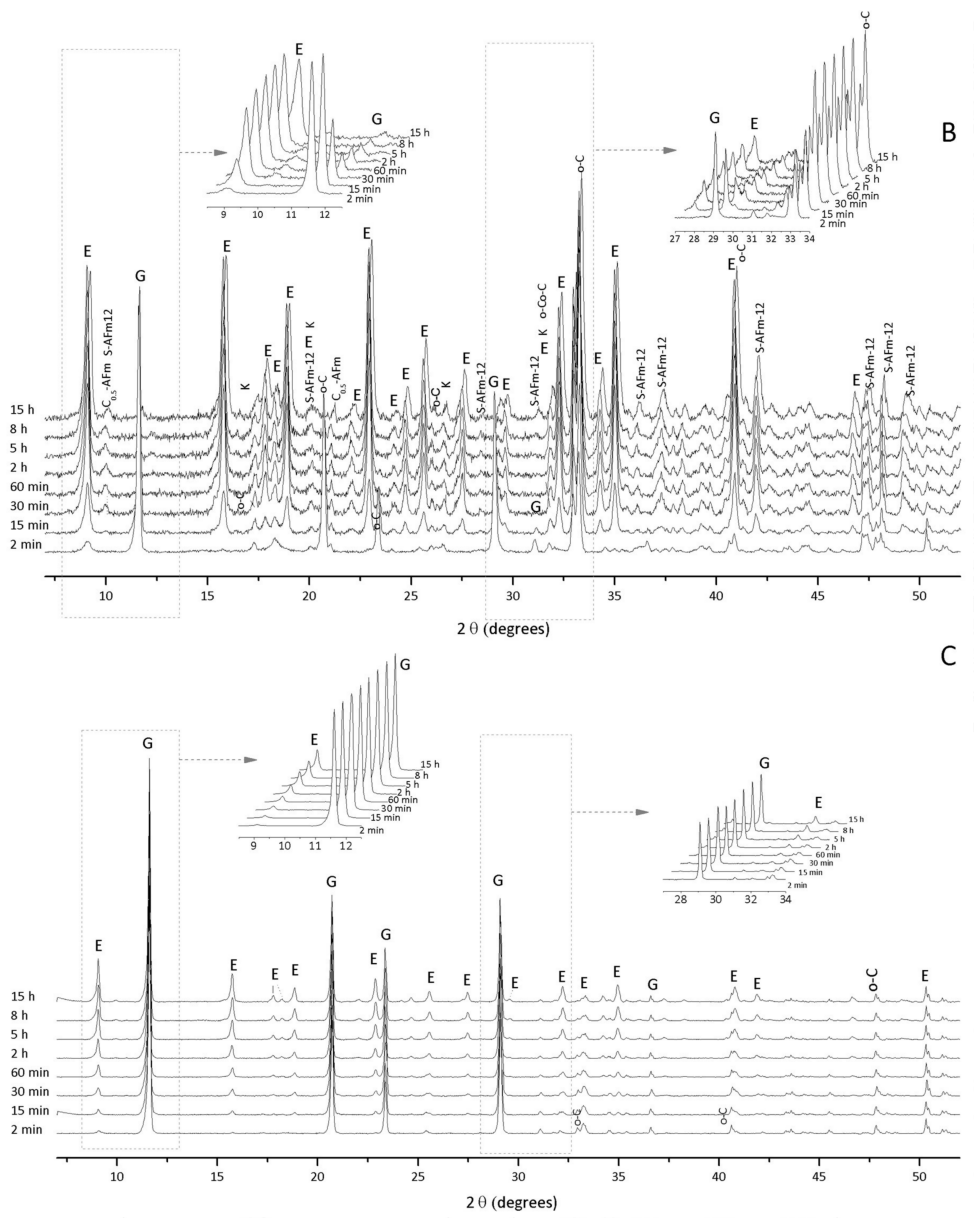
In situ XRD results of orth-C_3_A and gypsum hydration at gypsum/orth-C_3_A ratios of (**A**) 0.20; (**B**) 0.60; and (**C**) 1.90. o-C = orth-C_3_A, E = ettringite, G = gypsum, OH-AFm = C_4_AH_19_; k = katoite (C_3_AH_6_), C_0.5_-AFm = calcium hemicarboaluminate hydrate; $\bar{S}$-AFm = monosulfoaluminate.

Similar results are found for the orth-C_3_A and gypsum hydration systems (Figure 4) with ettringite formed over the full range of gypsum/orth-C_3_A ratios and $\bar{S}$-AFm precipitation favored at lower gypsum content. The consumption of the C_3_A during the hydration is higher for the orthorhombic-type structure regardless the gypsum content (except for the sample with no gypsum added), and the intensities of their characteristic peaks are lower compared to the corresponding systems based on cub-C_3_A. Furthermore, $\bar{S}$-AFm is more stable in the orth-C_3_A system, as it is also identified at a gypsum/orth-C_3_A ratio = 0.20 and 0.60. This phase is again produced as the gypsum peaks decrease from major to minor intensities, which occurs after five hours in the gypsum/orth-C_3_A = 0.20 sample and after 30 min in the gypsum/orth-C_3_A = 0.60 sample. The gypsum/orth-C_3_A = 0.60 system (Figure 4B) exhibits the highest reactivity degree due to a more pronounced decrease in the gypsum peaks. Unhydrated C_3_A appears before 20 min of hydration with almost complete hydration after roughly eight hours, and a higher and progressive formation of ettringite is clearly identified. Total gypsum consumption was not observed in the gypsum/orth-C_3_A = 1.90 sample, and this was the only sample in which the orth-C_3_A fully reacted within the first few minutes of hydration. In the presence of sulfates, the reactivity of C_3_A increases at a higher content of Na^+^ in its structure, and therefore, the orthorhombic phase is a more reactive polymorph when dissolved sulfate ions are present. The diffraction peaks for ettringite appear after two minutes of hydration in the orth-C_3_A samples at gypsum/orth-C_3_A ratios of 0.20, 0.60, and 1.90. The ettringite peaks in the gypsum/orth-C_3_A = 0.60 and 1.90 samples are more prominent than in the X-ray diffractograms for the cub-C_3_A samples. Unlike the sample, gypsum promotes the accelerated consumption of orth-C_3_A during the reaction due to the higher solubility of their Al_6_O_18_^18−^ ring structures in the presence of a sulfate source compared to that of the cub-C_3_A samples [34]. This finding is also corroborated with the slower cub-C_3_A consumption regardless of the smaller gypsum content during 15 h of analysis.

**Figure 5:**
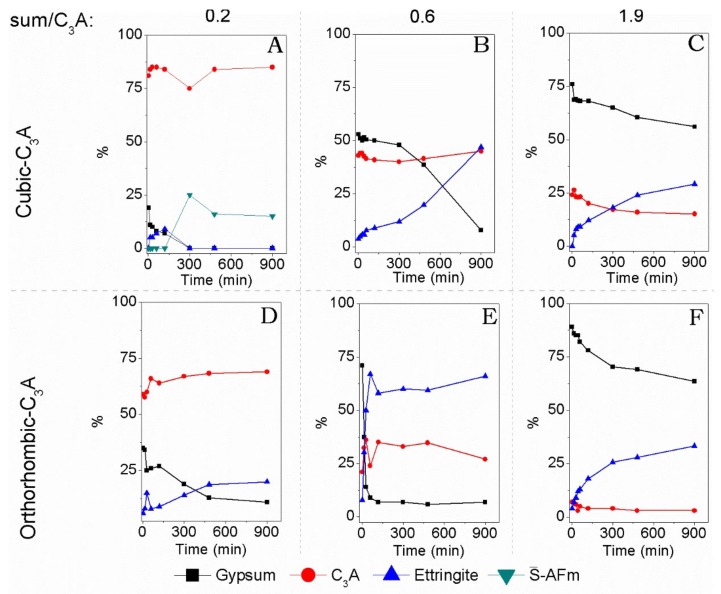
Reference intensity ratio (RIR) analysis of cub-C_3_A hydration with gypsum/C_3_A ratios of (**A**) 0.20; (**B**) 0.60; and (**C**) 1.9, and orth-C_3_A hydration with gypsum/C_3_A ratios of (**D**) 0.20; (**E**) 0.60; and (**F**) 1.9.

**Figure 6:**
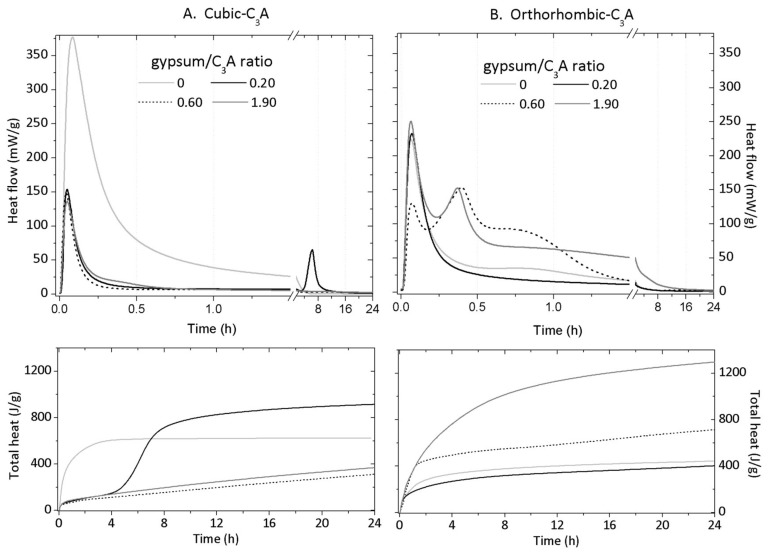
Rates of heat evolution (**top**) and cumulative heat (**bottom**) for (**A**) cub- and (**B**) orth-C_3_A hydration in the presence and absence of gypsum.

Figure 5 presented the RIR analysis using the major diffraction peaks for gypsum, orth-C_3_A, cub-C_3_A, ettringite, and $\bar{S}$-AFm. Figure 5A shows coherence between the minimum value for C_3_A and maximum of monosulfate, in 300 min (five hours), its formation was favored by the very low gypsum content. The levels of C_3_A and monosulfate remain constant, with no increasing in the reaction. There is also consistency in the increase and then decrease in the ettringite content, and a steady decrease in the gypsum content. This can be correlated to the cumulative heat of this sample (Figure 6A) in the IC test, where the image shows an abrupt change in the amount of the cumulative heat after five hours. Figure 5E indicates that the hydration of orth-C_3_A is fastest in the gypsum/orth-C_3_A = 0.60 system. The diffraction peaks for orth-C_3_A are not identified after 15 min of hydration in the gypsum/orth-C_3_A = 1.9 sample (Figure 5F), indicating that it is consumed faster than cub-C_3_A in its counterpart system at the same gypsum content (Figure 5C). The presence of amorphous calcium aluminate hydrates and some AFm-type structures cannot be reliably quantified by conventional XRD due their short-range ordering and is identified only through the deviation in the baseline between 15 and 25 degrees.

In summary, the XRD measurements showed that gypsum accelerates the consumption of orth-C_3_A more than cub-C_3_A during hydration. Stephan and Wistuba [11] studied the hydration behaviour of C_3_A solid solutions with MgO, SiO_2_, Fe_2_O_3_, Na_2_O and K_2_O. The authors found out that the hydration of these materials in the presence of CaSO_4_ was accelerated when C_3_A was doped with K_2_O or Na_2_O, whereas Fe_2_O_3_ strongly retarded the hydration.

### 3.3. Isothermal Conduction Calorimetry

The rate of heat evolution measured by IC depends greatly on the C_3_A crystal structure and gypsum content (Figure 6) with up to three exothermic peaks identified in each curve. For both cub- and orth-C_3_A samples, the first peak with maximum at roughly five minutes corresponds to the initial wetting and dissolution including the precipitation of OH-AFm and C_3_AH_6_ (at gypsum/C_3_A content of 0 and 0.20) and ettringite (at gypsum/C_3_A content of 0.60 and 1.9). The formation of these solid phases have been reported for the more dilute cub-C_3_A samples (w/s = 4, 10, and 25) [5,6,28,32] and pastes (w/s = 1) [8] and agrees with the previous results.

The addition of gypsum to the cub-C_3_A sample decreases the intensity of the main heat evolution peak (376.8 mW/g) to an approximately constant peak value (135.9 mW/g), irrespective of the gypsum content. This result is consistent with previous research [5], where the time of the highest heat release identified is unaffected by the gypsum content once present. However, the occurrence of the secondary peak, attributed to $\bar{S}$-AFm formation and consumption of initially precipitated ettringite, depends on the gypsum content. This peak occurs in the results for the gypsum/cub-C_3_A = 0.20 sample only at roughly six hours of hydration (Figure 6). This peak originates from the renewed hydration of cub-C_3_A that occurs once gypsum has completely dissolved. Recent research suggests that this renewed hydration of cub-C_3_A coincides with the desorption or consumption of Ca-S complexes, which adsorb onto the cub-C_3_A surface and inhibit the dissolution of this phase at earlier hydration times [39]. In the XRD result for the gypsum/cub-C_3_A = 0.60 sample (Figure 3B), peaks with very low intensities attributed to $\bar{S}$-AFm can be seen after 15 h. The low intensity of the $\bar{S}$-AFm peaks in the XRD results for this sample suggest the presence of a very low content, and the quantity of this phase formed (at this time) is quite low, and insufficient heat is measured by the equipment. In contrast to the cub-C_3_A system, adding gypsum to the orth-C_3_A hydration does not decrease the intensity of the initial exothermic peak (~220–250 mW/g), except for the gypsum/orth-C_3_A = 0.60 sample. The intensity of the initial heat release peak for the gypsum free orth-C_3_A sample is ~40% lower than that identified for the corresponding cub-C_3_A sample, indicating that orth-C_3_A is less reactive than cub-C_3_A in water, which agrees with [11,40] and the XRD results.

The addition of gypsum to the orth-C_3_A system does not greatly change the heat evolution rate at a gypsum/orth-C_3_A ratio = 0.20 with its main exothermic peak ~51% higher than that of the corresponding cub-C_3_A system. A prominent second exothermic peak is identified at ~23 min in the orth-C_3_A samples synthesized at gypsum/orth-C_3_A ratios of 0.60 and 1.90. This second peak coincides with the consumption of gypsum and precipitation of ettringite in the gypsum/orth-C_3_A = 0.60 sample. However, in the gypsum/orth-C_3_A = 1.90 sample, the amount of gypsum stabilized after the first few minutes of the reaction; therefore, this second peak can be attributed to the consumption of orth-C_3_A and the precipitation of ettringite. The higher heat released before 8 h in the gypsum/orth-C_3_A = 0.60 sample (Figure 5B) can be assigned to $\bar{S}$-AFm formation and mainly to the continuous formation of ettringite at the expense of gypsum; as is shown in the XRD results in Figure 3B. This result indicates that the hydration of orth-C_3_A occurs faster than cub-C_3_A in the presence of gypsum, which is consistent with existing research on the cub- and orth-C_3_A pastes in the presence and absence of lime [28,30,32].

## 4. Conclusions

This paper presented IC and in situ XRD analyses to follow the hydration of cub- and orth-C_3_A hydration in the absence and presence of gypsum. The results showed that orth-C_3_A reacts faster than cub-C_3_A in the presence of gypsum with early ettringite formation and gypsum and C_3_A consumption occurring in the former system. However, the hydration rates of cub- and orth-C_3_A still depend on the gypsum content. According to the XRD results, gypsum is consumed faster in the orth-C_3_A systems, mainly for an orth-C_3_A/gypsum ratio of 0.60. In the absence of gypsum, cub-C_3_A was found to dissolve faster than orth-C_3_A, releasing higher quantities of heat and producing calcium aluminate hydrate phases earlier. The different effects of gypsum on the hydration of both cub- and orth-C_3_A have important practical implications, indicating that the crystal structure type and quantity are key parameters to consider in optimizing calcium sulfate addition to PC clinker to ensure good workability throughout placement.

## Figures and Tables

**Figure A1:**
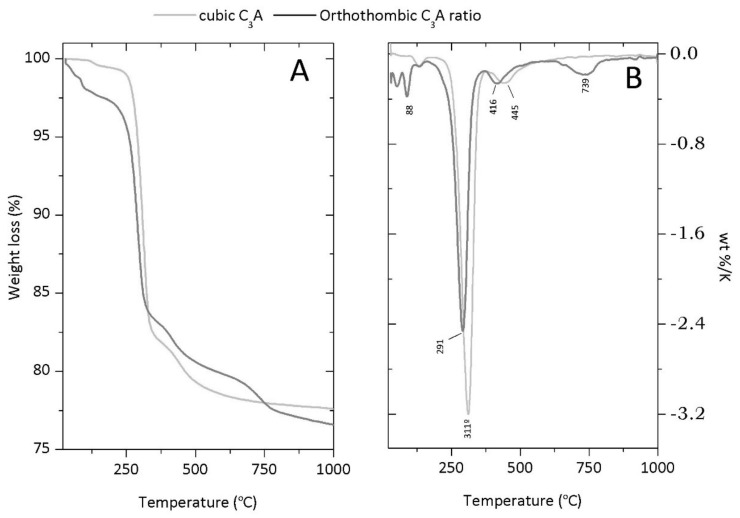
Weight loss curve profiles determined by (**A**) TGA and (**B**) DTG of hydrated pastes of Cubic and Orthorhombic samples without gypsum after three days.

**Figure A2:**
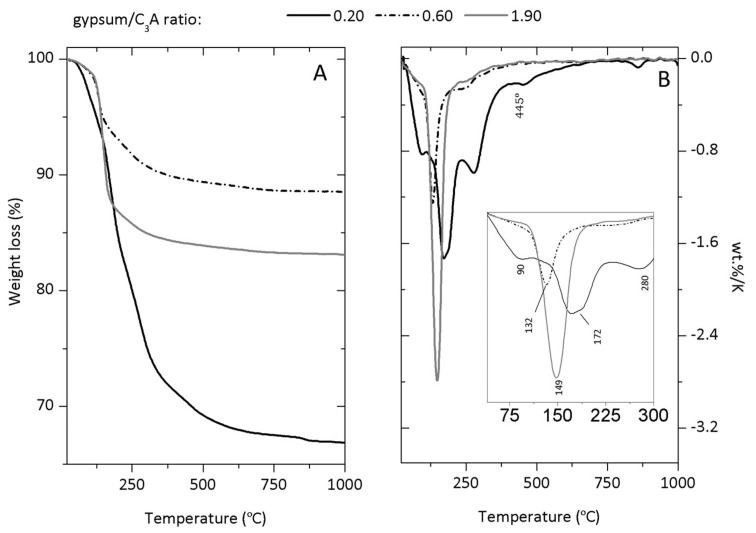
Weight loss curve profiles determined by (**A**) TGA and (**B**) DTG of the effect of different gypsum contents on cubic C_3_A pastes.

**Figure A3:**
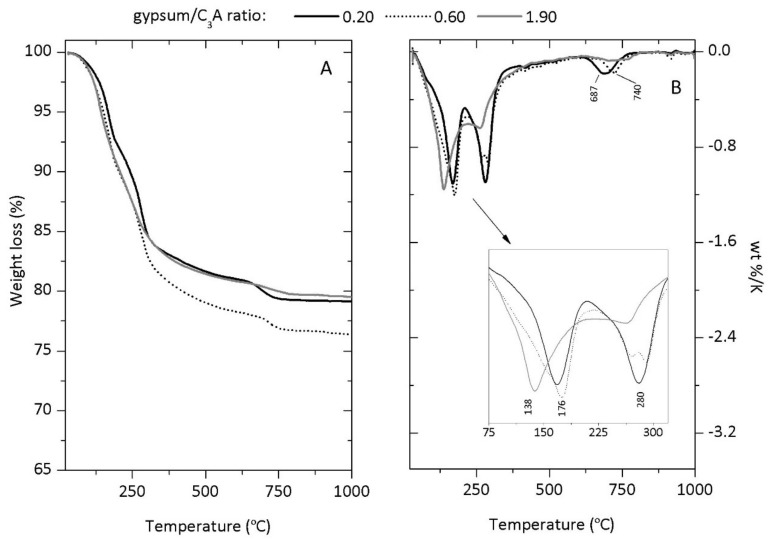
Weight loss curve profiles determined by (**A**) TGA and (**B**) DTG of different gypsum contents on orth-C_3_A pastes.

## Appendix A. Cement Chemistry Notation

- C: CaO
- S: SiO_2_
- Al: Al_2_O_3_
- H: H_2_O
- $\bar{C}$: CaCO_3_
- $\bar{S}$: SO_3_
- OH-AFm_19_: C_4_AH_(7+x)_ (or OH-AFm_(7+x)_)
- C_0.5_-AFm: Ca_4_Al_2_O_6_(CO_3_)_0.5_·11.5H_2_O
- $\bar{S}$-AFm: Ca_4_Al_2_O_6_(SO_4_)·14H_2_O
- $\bar{C}$-AFm: 3CaO·Al_2_O_3_·CaCO_3_·11H_2_O

## Appendix B. Thermogravimetric Analysis (TGA)

An analytical thermobalance model STA 409 PG (NETZSCH, Selb, Germany) under a flow rate of 60 mL/min of nitrogen and a heating rate of 10 °C/min up to 1000 °C was used. The unreacted C_3_A samples (cubic and orthorhombic) were assessed, which showed 1 wt % and 8 wt % mass loss on firing between 250 °C and 300 °C (aluminates hydrate) [41] and 650 °C (carbonates) by Thermogravimetric Analysis (TGA), consistent with the Rietveld analysis results.

To support the IC tests, the same hydrated C_3_A pastes samples assessed in the IC experiments were frozen at −196 °C with liquid nitrogen after being in the calorimeter for three days, kept in a freezer at −27 °C for 24 h, lyophilized for 16 h, and stored in a desiccator until analysis.

The TGA results (Figure A1) show a slight difference in the total weight loss (roughly one percent) between the cubic system and the orthorhombic system in absence of gypsum; where the hydration rate after three days of hydration can be comparable. Similar results are reported in Boikova et al. [40]. The free water evaporation is responsible for the presence of small peaks at temperatures below 100 °C. The derivative thermogravimetric analysis (DTG) results for both pastes show a strong peak between 291 °C and 311 °C due to the dehydroxylation of aluminate hydrated-type products, including OH-AFm_19_, C_3_AH_6_, and AH_3_ (at temperatures between 300 °C and 360 °C) [42,43]. The greater mass loss at approximately 300 °C for the cubic C_3_A sample (which exhibited a peak in the DTG with higher intensity) than for orthorhombic C_3_A indicates greater amount of aluminate hydrated-type products in the cubic C_3_A (Figure A1). These results obtained from DTG are consistent with the results previously presented for other analytical techniques (mainly the total heat released in calorimetry) where the cubic C_3_A in absence of gypsum exhibits a higher reactivity degree. Therefore, the DTG and XRD results for cub-C_3_A hydration in the absence of gypsum exhibit a higher dissolution rate and reaction than the analogous orth-C_3_A system. The orth-C_3_A paste shows a peak located at 739 °C, corresponding to decomposition of carboaluminate-type products (C_0.5_-AFm as was previously identified by XRD at early ages).

At lower gypsum contents (0.20), the profile of the DTG curve presents three peaks between 90 °C and 280 °C. The first peak occurs at ~90 °C, which is associated with free evaporable water, the second peak at ~170 °C, along with the other peaks at 90 °C and 280 °C can be attributed to $\bar{S}$-AFm [44]. Rheinheimer et al. [33] assessed scanning transmission X-ray microscopy (STXM) images and near edge X-ray absorption fine structure (NEXAFS) spectra of the gypsum/cub-C_3_A blends at a ratio of 0.20 after three days of curing, showing that this system stabilizes $\bar{S}$-AFm as the dominate precipitate, with only a small amounts of ettringite formed.

The inclusion of gypsum modifies the kinetic of reaction (as was shown in IC), as well as the type of products formed (Figure A2). The gypsum/cub-C_3_A ratios of 0.20 paste showed the highest total mass loss (33.2%) for cubic C_3_A systems. Higher contents of gypsum (gypsum/cub-C_3_A ratios of 0.60 and gypsum/cub-C_3_A ratios of 1.90) reduce the total mass of weight by a factor of roughly two compared to its corresponding system based only with cubic-C_3_A (gypsum/cub-C_3_A ratios of 0.0), which elucidates a clear retarding effect and lower content of hydrated products formed due to the presence of gypsum. This retarding effect was also identified through the reduction of total of heat released by these systems. DTG results for cubic C_3_A in the presence of gypsum show one mass loss peak between 100 °C and 270 °C with a maximum intensity between 100 °C and 220 °C which correspond to the loss of hydrated water of ettringite (~140 °C) and mainly gypsum (~150–190 °C) [45,46]. The intensity of this peak is clearly related with the gypsum content where gypsum/cub-C_3_A ratios of 1.90 shows a mass loss of 12.6% followed by gypsum/cub-C_3_A ratios of 0.60 which reported a value of 6.3%. DTG results for samples with higher amount of gypsum show strong mass loss peaks at 132 °C and 149 °C, which correspond to the loss of hydrated water in ettringite [45,46], and gypsum decomposition in the system with higher content of sulfates (1.90). The characteristic double peak of gypsum corresponding to the decomposition from dihydrate to unhydrate (at temperatures between ~150–160 °C and ~190–200 °C) is not observed and might be overlapped by the dehydration of the other products.

The orthorhombic systems with gypsum (Figure A3) exhibited a different behavior. The differences of total mass of weight loss between the orthorhombic pastes are not higher than 15%. DTG curves for orthorhombic-C_3_A show three peaks whose features depend on the gypsum content. Systems with lower gypsum content (0.20) showed a combined formation of $\bar{S}$-AFm due to the presence of two peaks located at ~175 °C and ~280 °C [44]. Considering the same authors, the peak located at 138 °C for the gypsum/orth-C_3_A = 0.60 and 1.90 can be attributed to ettringite. The orth-C_3_A samples, regardless of gypsum content, show a set of peaks between 650 °C and 780 °C, which reveals a higher susceptibility to carbonation. The XRD results are aligned with the results identified in TGA, where low contents of gypsum AFm-type phases are identified (peaks in the DTG at temperatures around ~180 °C and 280 °C). As the content of gypsum is higher regardless the type of C_3_A (orthorhombic or cubic) the ettringite is identified as a main hydrated product (intense peak around ~140 °C).
